# The Effect of Network Solvation on the Viscoelastic Response of Polymer Hydrogels

**DOI:** 10.3390/polym9080379

**Published:** 2017-08-19

**Authors:** Jan Zidek, Eva Kulovana, Josef Jancar

**Affiliations:** 1 Central European Institute of Technology, Brno University of Technology, Purkynova 123, 61200 Brno, Czech Republic; jancar@fch.vutbr.cz; 2 Faculty of Chemistry, Brno University of Technology, Purkynova 118, 61200 Brno, Czech Republic; xckulovana@fch.vutbr.cz; 3 SCITEG, a.s., 61600, Brno, Czech Republic

**Keywords:** hydrogel, deformation, solvation, molecular dynamics, water

## Abstract

The majority of investigations consider the deformation response of hydrogels, fully controlled by the deformation behavior of their polymer network, neglecting the contribution caused by the presence of water. Here, we use molecular dynamics simulation in an attempt to include the effect of physically bound water via polymer chain solvation on the viscoelastic response of hydrogels. Our model allows us to control the solvation of chains as an independent variable. The solvation of the chain is independent of other factors, mainly the effect (pH) which interferes significantly in experiments. The solvation of hydrophilic chains was controlled by setting a partial charge on the chains and quantified by the Bjerrum length (BL). The BL was calculated from the partial electric charge of the solvent and macromolecular network. When the BL is short, the repulsive Van der Waals interactions are predominant in the vicinity of macromolecules and solvation is not observed. For a long BL, the water molecules in the solvation zone of network are in the same range as attractive intermolecular forces and the solvation occurs. The model also allows the consideration of molecules of water attached to two chains simultaneously, forming a temporary bridging. By elucidating the relations between solvation of the network and structural changes during the network deformation, one may predict the viscoelastic properties of hydrogels knowing the molecular structure of its polymer chains.

## 1. Introduction

The hydrogel materials have a wide range of applications, for example, in medicine, cosmetics, and the food industry. Their advantage is an ability to control their physical properties in a simple way: a change of pH, degree of swelling, salt concentration or temperature [1,2,3]. The mechanisms of temperature and pH responsive rheological behavior of amphiphilic hydrogels have been known for a long time. The viscosity and deformation resistance are based on the formation of supermolecular structures such as micellar structure [1].

The next alternative structural factor is the solvation of chains [4]. It is the ability of macromolecular chains to uptake water or other solvents. It is a finer regulation of the properties than the above-stated factors. The advantage is that solvation does not influence the properties of hydrophobic parts of hydrogels. Even the modification of hydrophilic chains can be a significant factor. The mechanical response of materials with different solvability can often be various [4,5].

One of the limitations of the most experimental studies is that the effect of solvation cannot be distinguished from the other effects [6,7]. The main effect interfering with the solvation is acid-basic equilibrium and the formation of physically crosslinked networks. The real materials in a laboratory are mostly ionic gels, where both electrostatic potential and pH responsivity change together. We present a model study of the solvation and deformation properties. It is based on a model, where the effect of pH can be set independently of solvation. We can change only the effect of the solvation of hydrophilic macromolecules and keep constant the effect of micellar structure. Then one can observe systematically the change of deformation response with the increasing solvability of chains and find the structural interpretation of the solvation. The range of deformation properties of amphiphilic gels depends on the presence or absence of interacting groups and their distribution on the molecule [8,9]. The solvation properties can support self-assembly [10]. The influence of the solvent on the conformation of chains can be more complex. Schiessel [11] presented phase diagrams, where conformations of chains depend on electrostatic forces and type of solvent (good, theta, poor). The phase diagrams are valid for a dilute solution of chains, which are not entangled. However, they show information about the response of macromolecular chains with respect to solvation. In general, we can consider our model system as a polymer with a good solvent because the interaction of water with a segment of poly(ethylene glycol) (PEG) chains is larger than the interaction of PEG-PEG. We can expect that the chain will constitute ideal thermal blobs when the electrostatic interaction will be small. With increasing electrostatic interactions, the chain will be slightly stretched and the size of blobs will decrease. In the limit case, the conformations can be transformed to the ideal statistical chain.

The hydration in the model and the real material is regulated by the density of partial charge on the chains. The relation between hydration and charge on chains is theoretically described by the theories of polyelectrolytes. The basic parameter is a Bjerrum length (BL), which is the distance at which the electrostatic interaction between two elementary charges is comparable in magnitude to the thermal energy. The simply charged macromolecules were described by Manning [12]. He calculated the distribution of neutralizing counter-ions surrounding the charged macromolecular chain approximated as a straight charged cylinder.

Next, the influence of the partial charge on the flexible chains was described using the scaling theory of polyelectrolyte solutions. In the presented model, the electrostatic properties are related to the persistence length of polyelectrolytes. A steep change of behavior with increasing concentration of polyelectrolyte is observed [13,14]. The transition is from the macromolecular solution to the entangled network and it is observed as an increase of the viscosity of the solution.

Most of the theories of polyelectrolytes describe chains, which are charged by a full charge (positive or negative). The result is a distribution of solvent and counter-ions. The distribution of the solvent in the electrolyte was also investigated using molecular models [15,16]. Theories of macromolecular polyelectrolyte describe the formation of a double layer around the chains with a given sign of charge. Our model cannot form a double layer. The chains in our model are macroscopically neutral and have only partial charges with both positive and negative signs. The distribution of solvent is determined by electrostatic interactions with chains. The partial charge on the chains is compensated by water, which is also formally a neutral molecule, but it has both partial negative and partial positive charges (SPC-E model of water: [17]).

The model analysis of solvation was performed using model materials of the same composition but with different distributions of interacting groups [18]. The block and random poly-*N*-vinyl-2-pyrrolidone-*co*-2-hydroxyethyl methacrylate were compared. The hydrogels have the same composition but different solvation. The deformation response was a function of group distribution. Relatively more water molecules participated in solvating monomeric units in the random sequence hydrogel than those in the blocky sequence hydrogel. In the experiments, the hydration properties were regulated using a mixed solvent [19]. Mixtures of water with dimethyl sulfoxide and 4-Methylmorpholine N-oxide were compared. Both solvents have similar values of the Mark-Houwink exponent and their influences on the viscosity are equal. They differ only in hydration properties. Nevertheless, both the hydrogels have significantly different viscoelastic behavior and viscosity.

The mechanism of electrostatic interaction of an ionic solvent and macromolecular chains has been described in the literature. The common model of ionomer crosslinks are ions bridging the macromolecular chains [20]. The common example of such a crosslink is the alginate with Ca^2+^ ions, where the ionic parts of chains create strong bridges between macromolecular chains using ions. They have a strong influence on the viscoelastic properties. The strength of ionic crosslinks depends on the ion valence. Networks with bivalent and trivalent ions have higher hysteresis than monovalent ions [21]. In our model, the molecule of water has only a partial charge on oxygen (0.85 of elementary charge-e). It is not sufficient to create strong ionic crosslinks, but there can be expected some effects on deformation behavior. The real behavior of water in hydrogels has still not been investigated specifically. It was analyzed by relaxometric nuclear magnetic resonance (NMR) that the hydrogel contains three types of water: free, weakly attached and strongly attached [4,5]. Each type of water can be distinguished by its relaxation time in the spin relaxation NMR. The understanding of the relaxation and the viscoelastic response requires, among others, answering the question of the acting mechanism of all the types of water molecules. The water usually interacts with hydrophilic domains [22], hydrate functional groups of organic matter, or get trapped in hydrophobic microvoids. Moreover, water can modify the location and conformation of individual macromolecular segments [23].

The model enables us to observe the contribution of water to the deformation of the network. In our model, we cannot distinguish all types of water. We can only classify the water only as free and attached to the macromolecule. In our recent study [24], we described the influence of covalent crosslinks. We have quantification criteria for the intensity of covalent crosslinks and the deformation response. The criterion is a ratio: numbers covalent crosslinks/number of physical crosslinks (cpc). That enables us to observe the change of character of deformation with increased intensity of interaction.

We test the hypothesis that the water molecule can form a certain bridge. The water molecular bridges are described, for example, in the structure of the soil. The number of water bridges can be a secondary structural factor derived from solvation. The concentration of water bridges relative to physical nonbonding interaction can be a parameter, which can be in relation to the deformation response of the network. We have reported the materials with additive interactions of another type: additional covalent crosslinks to the physical network. Bridge from water molecule has a different effect on the deformation behavior as the covalent crosslink. From the model, we can compare the numerical intensities of both types of interactions: water bridge and covalent bond.

## 2. Models and Methods

### 2.1. Molecular Model

**Figure 1:**
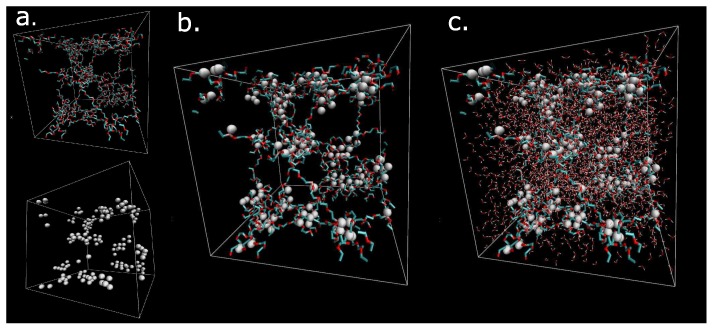
Visualization of the dry network of network structure; (**a**) white- physical crosslinks; cyan-red chains hydrophilic PEG chains; (**b**) Physical network from components from figure a; (**c**) solvated box. Box is cubic with side 5 nm.

The model is based on an existing minimalistic model of physically crosslinked hydrogel [25]. The model mimics the real chemical structure of poly(ethylene glycol) (PEG) gel micelles endcapped with Acrylic Acid (AA). Parameters of the model are summarized in Table 1. The model structure includes clusters, representing simplified micelles, which are formed by interaction energy of acrylic acid. The formation of physical crosslinks is independent of the solvation of chains. The formation of micelles is controlled by the AA interacting groups, which have strong mutual physical interactions. They are responsible for formation of micelles (Figure 1). The micelles are connected by the flexible chains of poly(ethylene glycol) with sequence (CH_2_–CH_2_–O).

A primary sequence of the model is a repeating unit of 10 atomic groups: –[(CH_2_–CH_2_–O)–(CH_2_–CH_2_–O)–(CH_2_–CH_2_–O)–AA]–. Each chain contains 20 repeating units. They are periodic molecules and they are artificially slightly entangled. The last residue of the chain is covalently bound with the first residue of the periodic image of the same chain. The model network contains 16 entanglements in order to prevent the separation of individual chains from the others. Each chain must be entangled with two other chains. The chains were not connected by covalent bonds. The atomic groups in chain are connected by harmonic bond potential with strong force constant (300,000 kJ·mol^−1^·nm^−2^) in order to keep the rigid bond. As well the angles were modeled as a harmonic oscillator with relatively small force constant 480 ·mol^−1^·deg^−2^. The angles can be stretched when the chain is subject to tension. The dihedral angles were not applied. The model was designed to mimic the behavior of freely rotating chain.

A secondary structure was previously based on interactions in the model. The basic set of interactions is included by GROMOS force field: the nonbonding interactions of CH_2_ and O. The AA group was user defined. Its mass was 72 (molar mass of acrylic acid) and the interaction between two acrylic acids was set as Lennard Jones potential with energy gap 25 kJ·mol^−1^. It is energy comparable to the energy of hydrogen bonds. The charge of AA group was set 0, and the other charges were variable. The interaction of AA with other groups (CH_2_ and O, oxygen from water and hydrogen from water) was numerically set equal to the standard interaction between two CH_2_ groups in force field.

The network structure was formed from an initial configuration of entangled network without physical clusters. The interaction AA–AA groups was decreased to the level of other interactions. The network was solvated in the state of random network before formation of micelles. The solvation was performed by a procedure, which is part of GROMACS software package. Next, the interaction of AA–AA groups was increased to the value 25 kJ·mol^−1^ and the micellar structure was formed. There is formed mostly 19–21 clusters per simulation box. The number of physical clusters is constant during the deformation only the individual interacting groups can switch from one cluster to another one. During the simulation without deformation, the structure seems to be static. The clusters remain unchanged and the segmental hops of AA groups are not observed as well.

A structural analysis was based on cluster analysis. The AA group was added to the cluster when another AA group was found in distance ≤0.4 nm. Both the groups were present in the same cluster. Next groups were included in the cluster until they were in distance ≤0.4 nm cluster. The clusters can be composed partly of intramolecular and partly of intermolecular AA groups. The clusters were detected by cluster analysis algorithm, which is included in MATLAB software. The periodic structure of box was reflected by the cluster analysis.

The AA groups are important for the pH responsive behavior. Similar molecules are used for regulation of pH response in real hydrogels [2]. They can be defined in two variants (neutral R–COOH/dissociated R–COO–). Neutral is marked HAA and it has strongly attractive interaction. The dissociated molecule is marked as AA– and has repulsive interaction due to repulsion of two negatively charged groups. The ratio of attractive non dissociated and repulsive dissociated groups [AA–]/[HAA] is calculated from Henderson-Hasselbalch equation. We need to know the value of pKa (for acrylic or itaconic acid are presented in literature).

(1) $$pH=pK_{a}+\log\left(\left[AA–\right]/\left[HAA\right]\right)$$

In this article, we set acidic pH for all networks ([AA–]<<[HAA]). All AA interacting groups in the model are in attractive variant HAA (in this model the attractive interaction was set to 25 kJ·mol^−1^). That setting of pH is completely independent of the setting of hydration, which is regulated by other constants.

The solvability was regulated by a partial charge on the chain. The positive partial charge $\delta_{CH_{2}}$ is set on the methylene (CH_2_) group and a negative partial charge is set on oxygen $\delta_{O}=-2\delta_{CH_{2}}$. The total charge of the whole network must be zero. The partial charge on the (CH_2_) group was changed in a range from 0 to 0.5, whereas the charge on oxygen was changed from 0 to −1. The model box was saturated by water. In our models, we use SPC-E model of water with charges on water $\delta_{OW}=-0.85$ and hydrogens $\delta_{HW}=0.425$ .

The GROMACS software has a functionality of box deformation. That functionality enables one to deform the box in the principal strains $(\epsilon_{x},\epsilon_{y},\epsilon_{z},\gamma_{xy},\gamma_{yz},\gamma_{xz}),$ where ϵ is a normal strain in the direction of the principal axis and γ is a shear deformation. The deformation was applied to the boundary box in each molecular dynamics step. Individual atoms are shifted affinely to the boundaries of the model. Their position is then corrected by molecular dynamics simulation. The primary result of the molecular dynamics simulation is shear energy density.

The pure shear stress was calculated:

(2) $$\tau =\frac{\partial W}{\partial \gamma }$$

where *W* is shear energy density and (γ) is relative shear deformation. The τ is a shear stress. The input parameter for the shear deformation is a shear rate $\dot{\gamma }$. The deformation rate is $0.1\;{\mathrm{ns}}^{-1}$ (100% deformation in 10 ns).

The model shear rate is high in comparison with the real strain rates. Even the high strain rates reflect the real behavior of hydrogels because the structural changes in the model are induced by the deformation. We found that both deformation response and structural changes are quicker than in real material, but the relations between structure and deformation response will be similar to those in a real material. Such structural changes are in our model and are accelerated in comparison with real material structure. The evolution of material structure can be qualitatively compared to different samples. The model during deformation is not in equilibrium, and dynamic character can be expected. The dynamic deformation as a function of strain rate is not discussed in this article. All the simulations are investigated at one rate of deformation, where the dynamic effects are clearly observable. The deformation amplitude 100% was selected. The samples have a constant shear rate of 0.1 ns^−1^ constant amplitude of deformation: 100%. The shear strain was applied in *x* direction of the box. The deformation is set as a parameter of GROMACS. The strain rate was rate equal to 0.5 nm·ns^−1^.

The individual models differ in amount of electrostatic interaction.

### 2.2. Solvation of Macromolecules

The primary input parameter was a partial charge on the oxygen (O) of PEG, and the partial charge of CH_2_ was calculated in order to keep the total charge of simulation box electrically neutral. The deformation properties do not grow linearly with a partial charge. The parameter of partial charge was substituted by parameters of solvation of chain. The parameter of solvation is a number of molecules, which are in the solvation zone or in the vicinity of chains. The boundary of the solvation zone was delimited by the Bjerrum length. The Bjerrum length is the distance from the molecule, where the electrostatic potential is equal to the thermal energy (kT). The Bjerrum length can be calculated also from the partial charge:

(3) $$l_{b}=\frac{1}{4\pi \varepsilon_{0}\varepsilon_{r}}\frac{\delta_{1}\delta_{2}e^{2}}{kT}$$

where $\delta_{1}e$ and $\delta_{2}e$ are partial charges of network and solvent (*e* is an elementary charge); *kT* is thermal energy, and $\varepsilon_{0}$ and $\varepsilon_{r}$ are permittivity of vacuum and relative permittivity of solvent (water). The Bjerrum length is a distance where the attractive electrostatic energy is equal to the thermal energy. The non-bonding Van der Waals energy has a repulsive zone which prevents the collisions of atoms. Outside the circles is an attractive zone where the water molecules can be attracted to the macromolecular chains. The boundary between the attractive/repulsive zone and the Bjerrum length can be considered a criterion for whether the water can be present in the solvation zone.

## 3. Results

The aim of this article is to describe relations between the partial charge on the chain and the deformation response of the network. We present the set of simulations with the same structure and external conditions. The only variable parameter, which can be set independently, is the charge on a flexible macromolecular chain, particularly on oxygen from PEG $\left(\delta_{O}\right)$. The second parameter, charge on flexible chains on the methylene group from PEG is derived from the condition of macroscopic electrostatic neutrality of the macromolecular network. Other parameters of simulations, such as interaction energy for the formation of micelles, charge on water, and weak Van der Waals energy, are held constant for all simulations (Table 1). The mechanical properties of the hydrogel are mostly controlled by pH responsive behavior (see. Methods: Equation (1)). The setting of pH is constant for all networks in this article.

The macromolecular network is visualized in Figure 1 by means of VMD software (Theoretical and Computational Biophysics Group, University of Illinois at Urbana-Champaign, Urbana, IL, USA) [26]. The dry hydrogel network consists of components: network (Figure 1a, up) and physical crosslinks (Figure 1a, down). The addition of both components (network and physical clusters) creates the model of the dry hydrogel network (Figure 1b). The next addition of the solvent to the organic phase network result is in the complete hydrogel material structure (Figure 1c). The white clusters are cores of micelles. The cyan-red components are flexible chains composed of hydrophilic poly(ethylene glycol) chains. The small red-white objects in Figure 1c are molecules of water. The distribution and motion of water molecules are key properties, which are analyzed in this article. Figure 1 illustrates a constitution of the model. The secondary structure of the model was created by self-assembly of current size (box size = 5 nm) using molecular dynamics simulation.

**Figure 2:**
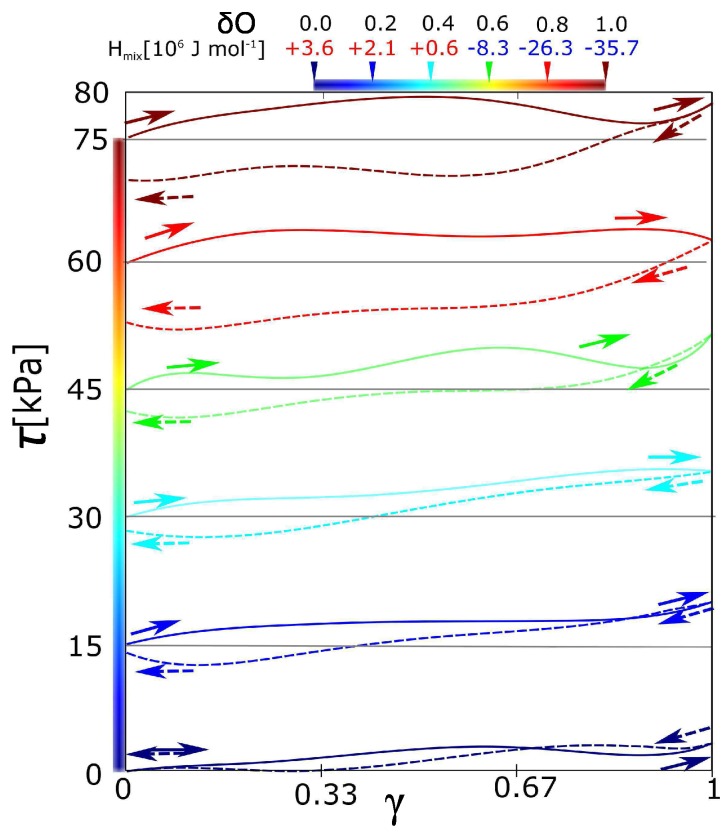
Stress-strain curves of forward-reverse shear deformations for networks with different partial charges. Charges on oxygen from PEG were $\delta_{O}$ = 0.0 to −1.0. Solid lines are forward deformation, dash lines are reverse deformation. All curves show the hysteresis. The curves for $\delta_{O}$ = from −0.2 to −1.0 were shifted equidistantly along the *y*-axis for better visibility. (Upper legend $H_{mix}$ is a mixing enthalpy of the individual networks.

The intensity of electrostatic interaction is given by a charge on the macromolecular chain. Specifically, the input parameter is a negative charge on oxygen in poly(ethylene glycol) monomer. Its range is from 0 to −1 of the elementary charge. We present value of mixing enthalpy in Figure 2 upper legend. Its sign gives the information about thermodynamic stabilization or destabilization by solvent. The positive sign means that the solvent destabilizes and negative sign means stabilization.

The main presumption is that the macroscopic properties are derived from the interaction of solvent and macromolecular chains. The variation of charge should affect also the both intra- and intermolecular electrostatic interaction between atoms of poly(ethylene glycol) chains. The influence of charge on the interaction in poly(ethylene glycol) was calculated.

The interaction can be detected by the models of dry network without presence of solvent. All the components of potential energy reflect the response of PEG chain or the interacting groups. The criterion is a difference between energy after and before deformation. The influence of charge on bond energy is almost negligible and influence on bond angle energy is small. The increase of nonbonding energy is comparable to the system with water. Only the term of electrostatic energy was different for the network with solvent and without solvent. The change of electrostatic potential energy for $\left(\delta_{O}\right)$ = −1.0 without solvent was 120 kJ·mol^−1^. On the contrary, the increase of electrostatic interaction with solvent is in range 2000 kJ·mol^−1^. The small electrostatic potential of dry network is composed only of 1440 positively and negatively charged groups. The network with a solvent contains 8781 charged atomic groups.

The next criterion is the electrostatic short range interaction, which is standard output of molecular dynamics simulation of solvated network. The short range interaction is a specific part of energy, which is observed in the range of cutoff ratio of nonbonding interactions (0.6 nm). The numerical criterion, whether the polymer solvent interaction plays a role, is the sensitivity of interaction to the change of partial charge on the chain. It is a difference of interaction for the charge $\delta_{O}$ = 0 and −1. Its value for water-water interaction is 6800 kJ·mol^−1^ for PEG-PEG -1780 kJ·mol^−1^ and interaction PEG-water is 41,200 kJ·mol^−1^. The polymer/water interaction is influenced by partial charge on chain more significantly than the binary interactions.

The next question is, whether the charge influences the secondary structure. It was found that the high charge: $\delta_{O}$ = −0.8 and $\delta_{O}$ = −1 decreases the size of physical crosslinks. The result is decreased fraction of physical crosslinks in entire simulation box. The charge does not have influence on a number of physical crosslinks. The decrease of cluster size does not have to change the viscoelastic properties.

### 3.1. Deformation Properties

The macromolecular networks with structure from Figure 1 were deformed by shear deformation. The deformation is accompanied by an increase of potential energy. The energy density has relation to shear stress (Equation (2)). If the sample is reversely deformed, the tension on the sample is mostly released. The reverse stress is not exactly the same as the forward one. This is due to dynamic structural changes, which are present in the network [24]. The surface between forward and reverse stress-strain curve is an energy density, which is lost during the deformation cycle.

All the stress-strain functions for the hydrogel with variable intensity of electrostatic interaction show certain hysteresis (Figure 2). All stress-strain curves start in shear stress = 0. The starting points of curves for a charge −0.2 to −1 are shifted equidistantly (with interval 15 kPa) in order to distinguish individual curves. It was observed, that hysteresis grows with the charge on the hydrogel network. The function of hysteresis on the charge was not linear. It seems that there is an unambiguously sharp difference in hysteresis between the curves $\delta_{O}$ = −0.4 and −0.8. The curves in the interval up to $\delta_{O}$ = −0.4 are similar in the low viscoelasticity. On the other hand, the curves starting from the charge $\delta_{O}$ = −0.8 are significantly viscoelastic and similar as well. The network with partial charge $\delta_{O}$ = −0.6 is ambiguous. It could be considered rather as a network without hysteresis.

**Figure 3:**
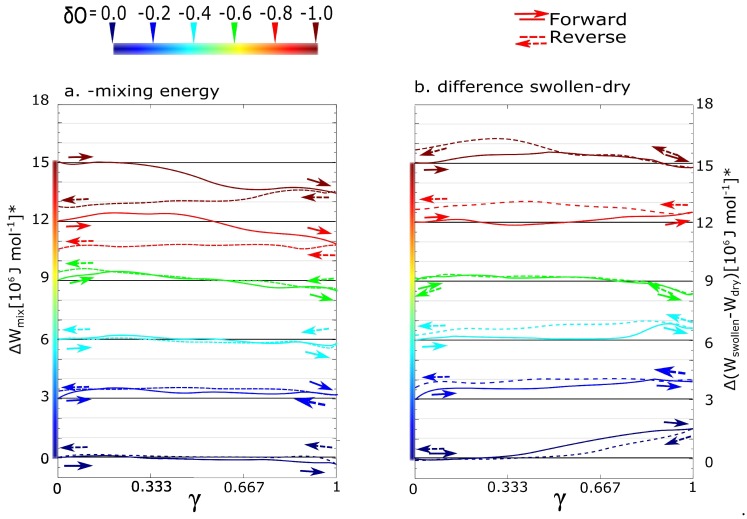
Contribution of solvent during forward-reverse shear deformations; (**a**) the components of mixing energy: The energy of dry network and solvent were separated from the total potential energy of swollen network. The physical meaning is a thermodynamic contribution of mixing energy during deformation; (**b**) mixing as a difference of two simulations: Swollen and dry networks. The physical meaning is the influence of water on the evolution of the network.

In order to find a more specific classification of the networks, the networks can be classified by the mixing energy derived from total energy density. The differences in function of mixing energy density between networks with low and high charges are more significant than in the case of stress-strain function. The $H_{mix}$ in previous Figure 2 means absolute value of mixing energy. The ΔW in Figure 3 means an increase or a decrease of energy density during deformation. It is the participation of mixing in the deformation. The contribution may be evaluated from two aspects (series a and b). Both the aspects are described by the same equation:

(4) $$\Delta W_{mix}=\Delta W_{gel}-\left(\Delta W_{ntw}+\Delta W_{solvent}\right)$$

where the values ΔW are partial energies.

In the case of Figure 3a, the partial energy densities were extracted as components of the total energy density of the swollen simulation box ($\Delta W_{gel}$). The partial energies are energy of the dry network ($\Delta W_{ntw}$) and the solvent ($\Delta W_{solvent}$). In that case, ($\Delta W_{ntw}$ and $\Delta W_{solvent}$ were calculated as components of total energy from the same simulation. The variant shows the thermodynamic stabilization or destabilization of the micellar structure.

In the case of Figure 3b, the partial energy densities were taken from individual simulations. The $\Delta W_{ntw}$ value of energy density was calculated from deformation of the dry network without solvent. In the same way, we deformed a box of pure water ($\Delta W_{solvent}$). However, the response of the solvent on deformation is zero in this case ($\Delta W_{solvent}$ = 0). The variant b. shows what the influence of water on the structural evolution of gel during deformation is. It is the information about how that deformation would have differed in the case of absence of a solvent.

According to the mixing energy, we can classify the network with $\delta_{O}$ = −0.6. Until the partial charge, the mixing contributions from Figure 3a,b are more or less equal. In that case, the effect of solvent is in thermodynamic stabilization or destabilization of the network. In the case of partial charge from −0.8 to −1, there is some additional effect of solvent. We performed the deformation functions and mixing energy (like in the Figure 2 and Figure 3) also for charges −0.1, −0.3 … to −0.9. They are not presented because all functions in intervals of charge from 0.0 to −0.5 have similar course. As well the functions in interval from −0.8 to −1.0 are similar. Instead of the deformation function of network with charge −0.7, we present below one with a partial charge −0.667. From the mixing energy, the qualitative change of network behavior is between the networks with charge −0.6 and small hysteresis and charge −0.7 with just significant hysteresis.

The input parameter is the average charge density in the network when the distribution of charge is more or less uniform. However, the average charge density $\delta_{O}$ in Figure 2 is not a sufficiently specific parameter. One variant is that the factor will be total charge density in general. The alternative interpretation is that the key factor is a presence or absence of some highly charged monomers, and the average charge density does not play a role. In this section, we investigate the difference in behavior of these two aspects. In the first case, we set the uniformly distributed partial charge. In the next case, we have set the same average partial charge; however, the highly charged monomers alternated with electrically neutral ones. We note that the model enables us to set the partial charge on the poly(ethylene glycol). The highly charged monomeric units were with $\delta_{O}$ = −1.0 and neutral monomers with charge $\delta_{O}$ = 0.0. The average partial charge was adjusted by their combination.

**Figure 4:**
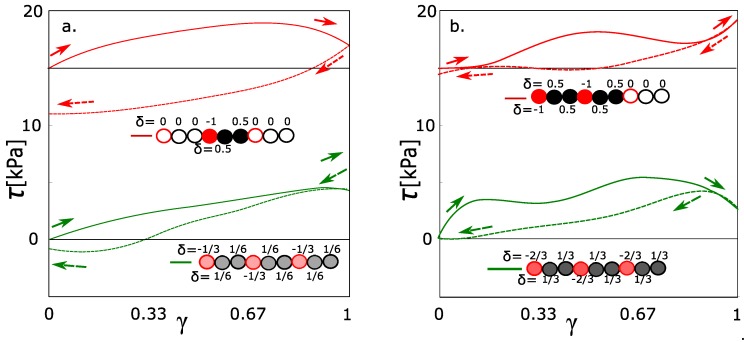
Stress-strain function. Green and red curves are with the same density of charge and different distribution: Green-charge uniformly distributed; red, the same average density as green, but the unity charge is accumulated on one monomer; (**a**) charge density 0.333 per monomeric unit; (**b**) charge density 0.667 per monomeric unit.

The box contains 480 monomeric units of poly(ethylene glycol). All monomeric units can be classified into the groups 160 charged/320 uncharged monomers. The number of charged 160 groups was selected in order to be comparable to the physically interacting groups (160) forming the micelles. Such combination gives the average charge $\delta_{O}$ = −1/3. The alternative model is where all monomers will be charged to the $\delta_{O}$ = −1/3 as a network with a uniform distribution of charge. The network with a uniformly distributed charge is equivalent to those functions calculated in Figure 2. The green stress-strain function in Figure 4a fits into the context of the previous simulations with charges −0.2 and −0.4. The red line in Figure 4a is a stress-strain function of a network with an unequally distributed charge. The hysteresis was observed in the network with an unequally distributed charge. It means that the highly charged groups must be present, even when the average charge density is relatively small.

Analogously, the same pair was modeled for average charge density −2/3 per one monomeric unit. From the green line in Figure 4b, the hysteresis is observed at the average charge density −0.667. In the special case, two monomeric units with a partial charge of −1.0 were alternating with one uncharged monomeric unit. The hysteresis was observed as well; however, we observed the decrease of hysteresis. It was a slightly unexpected result, which was reconfirmed by repeated simulations. For comparison, we would like to highlight that the deformation is not a monotonous function. It runs in waves (also the functions in Figure 2). Each wave means that we reached the actual limit stress of physical network and the physical network is relaxed. The limit stress for every network in this article is approximately 5 kPa. From this aspect, the red curve in Figure 4b behaves similarly like any other function from this article. The deviation from the normal course of functions is caused probably by a dynamic character of behavior. The increased stress on the physical crosslinks can lead to quicker relaxation.

### 3.2. The Structural Aspect of Deformation

In this section, we will find the structural aspect of network, which would be in correlation with deformation response.

**Figure 5:**
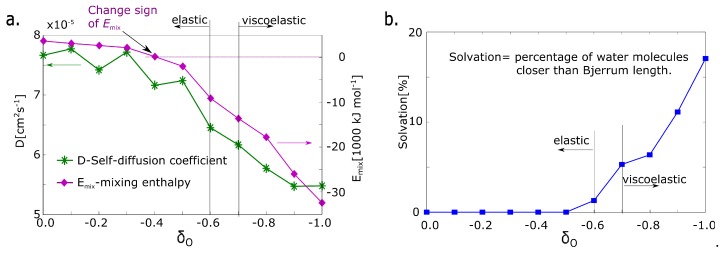
Structural parameters of hydrogel models. (**a**) $E_{mix}$: mixing energy calculated from simulation; D: self-diffusion coefficient of water; (**b**) degree of solvation—fraction of molecules, which are in the solvation zone (distance from macromolecular chains less than the Bjerrum length).

The first parameter is the absolute value of mixing energy in an undeformed model network. The sign of mixing energy is related to the physical quality of the network. It detects whether the solvent stabilizes or destabilizes the network thermodynamically. The sign is changed from positive to negative at the partial charge $\delta_{O}$ = −0.4 (Figure 5a). That structural parameter is not in correlation with deformation properties.

The next structural parameter is self-diffusion of water. The self-diffusion of water was calculated from molecular dynamics simulation. The value was fitted from the time function of the mean squared displacement of water molecules, which is the standard output of molecular dynamics simulation [27]. The self-diffusion coefficient decreases gradually with the decreasing value of the partial charge. The water molecule is immobilized with increased charge. However, a significant change between $\delta_{O}$ = −0.6 and $\delta_{O}$ = −0.8 is not observed.

The next property is the solvation of macromolecular network. The criterion of solvation is a presence in solvation zone limited by the Bjerrum length. The Bjerrum length calculated from Equation (3) was selected as a criterion of presence in the solvation zone. The number of water molecules in the solvation zone is almost zero up to a charge of $\delta_{O}$ = −0.5 and it is still very small at the charge of $\delta_{O}$ = −0.6. Starting from a charge $\delta_{O}$ = −0.7 it grows significantly (Figure 5b). The parameter changes in the same range as deformation response.

Next, the relation between partial charge and solvation was derived by the analytical model. The result is a percentage of water molecules in the solvation zone. The partial charge on the oxygen groups in PEG is a single independent input variable parameter of our numerical model. The charge on the methylene group (CH_2_) is related to the charge of the O group. The total charge of the model network must be zero.

(5) $$\delta_{O}=-2\delta_{CH_{2}}$$

The charge $\delta_{CH_{2}}$ is set as value $\delta_{1}$ to Equation (3).

The second partial charge ($\delta_{2}$, Equation (3)) is a charge on oxygen from water (OW). The OW in SPC-E model of water has a partial charge $\delta_{2}$ = −0.85. The solvent has constant properties for all simulations: charge ($\delta_{2}$), nonbonding interaction with chain and distribution of partial charge. The temperature is T=300 K. The $\varepsilon_{r}$ is relative permittivity of water for given temperature ($\varepsilon_{r}$ = 80). The other symbols are physical constants: permittivity of vacuum $\varepsilon_{0}$, Boltzmann constant *k*, and elementary charge *e*.

The total potential energy is a sum of Lennard-Jones potential energy and electrostatic potential.

(6) $$E=E_{LJ}+E_{el}$$

**Figure 6:**
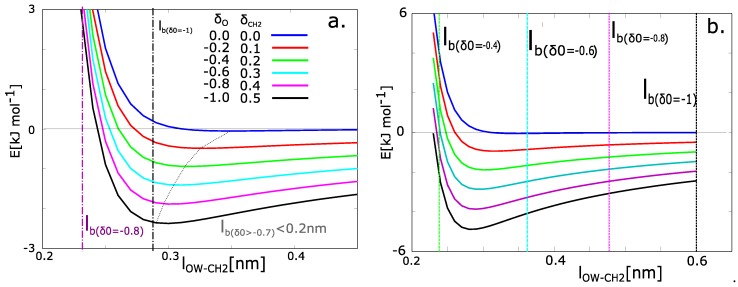
Potential function of the attraction of water to the macromolecular chains as a function of the distance of water from the macromolecular chain and partial charge (δ) on a CH_2_ group. (**a**) Results for macroscopic relative permittivity of water ($\varepsilon_{r}$ = 80); (**b**) results for relative permittivity of water on molecular level ($\varepsilon_{r}$ = 40); The potential energy is the sum of Lennard-Jones potential and electrostatic potential; $l_{b}$— Bjerrum length.

The parameter of Lennard-Jones potential is the depth of potential well ($E_{0}$) and equilibrium distance ($l_{m}$) at minimal energy:

(7) $$E_{LJ}=E_{0}\left[{\left(\frac{l_{m}}{x}\right)}^{12}-2{\left(\frac{l_{m}}{x}\right)}^{6}\right]$$

where *x* is an actual distance between the CH_2_ group in the PEG chain and oxygen from water ($l_{OW–C}$). The original Lennard-Jones potential energy is a blue curve in Figure 6. In our simulations, the potential energy has the function minimum at −740 J·mol^−1^ and equilibrium distance 0.45 nm. The function of potential energy was taken as the same as in simulations. The electrostatic potential is calculated from Coulomb’s law:

(8) $$E_{el}=\frac{1}{4\pi \varepsilon_{0}\varepsilon_{r}}\frac{\delta_{1}\delta_{2}\;e^{2}}{x}$$

where *x* is the actual distance of the atoms. The potential energies from Equation (6) with different charges are presented in Figure 6. The charged chains attract the molecules of water with higher energy and the distance is tighter than the chains with zero charge.

The relation between the charge and solvation can be calculated from the functions in Figure 6a. The Bjerrum length (BL) is plotted as a dashed vertical line on the *x*-axis. The BL contains the information of thermal energy. Next, the minimum of the function was found. The actual value of potential energy at the Bjerrum length was calculated ($E_{bl}$). Then the energy in the minimum of function was calculated ($E_{min}$). Let us consider the fact that the molecule of water will be some distance from the macromolecule. The most probable position is the distance with minimal energy. The positions in solvation zone have a barrier in the form of an increase of energy $\Delta E=E_{bl}-E_{min}$. The probabilities of existence of water in the solvated zone are given by partition functions: $Q_{inside}\propto exp\left(-\Delta E/kT\right)$, $Q_{outside}\propto exp\left(0/kT\right)$.

The probability of presence in the solvation zone was calculated from the partition functions. They can be evaluated numerically. In the case of our model, the networks with partial charges on oxygen (δO≤−0.6) have a near zero probability of the presence of water. For the partial charge of oxygen δO = −0.8 the probability that the water will be in solvation zone is 15%. For the partial charge δO = −1.0, the energy in the Bjerrum distance and minimal energy are very close. The states of water in the solvation zone and water in the attractive zone of Lennard-Jones potential are equivalent. From the energies, we can consider that 100% of water molecules have the tendency to persist in the solvated zone.

The analytical model in Figure 6a describes situation with many simplifications. For example, we use a macroscopic value for the model parameter: relative permittivity of solvent (symbol $\varepsilon_{r}$ in Equation (8)). The macroscopic tabulated value for water at 293.15 K is 80. The real permittivity on molecular level can be significantly lower than the macroscopic one. Itoh and Sakuma calculated the permittivity of water near the graphite surface can be a function of distance from surface [28]. The $\varepsilon_{r}$ decreased from macroscopic value up to 3.91. The reason for this decrease is the orientation secondary structure of water near the surface. The molecules of water molecules near the graphite surface are oriented along the graphite plane in contrast to the water molecules in bulk water. The orientation is one factor, which decreases the permittivity. Next factor is restricted formation of hydrogen bonds. The orientation of water molecules near the plane is not favorable for the formation of hydrogen bonds between two water molecules. It increases the mobility of water molecules near the surface and changes the electrostatic potential.

Our model has partly similar properties as model of Itoh and Sakuma, however, it differs in several aspects. The similarity is that the spatial distribution of water molecules near the solid network is different from water distribution in bulk water. One can imagine, that the formation of hydrogen bonds water-water would be hindered in the same way as in the model of graphite plane. That factor probably causes the decrease of relative permittivity. The different aspect of our model and the graphite model is the behavior of solid phase. The model of graphite plane has ideal planar surface, which is probably fixed in time. Our model water is in vicinity of flexible macromolecular chain, which moves during the simulation. Itoa and Sakuma calculated the value of relative permittivity 41.15 for the water molecules in perpendicular direction to graphite plane. That value could be suitable value for our model water. We calculated the same model from Equations (6)–(8) with relative permittivity 40 (Figure 6b). The results diverged from results from molecular dynamics simulation. According to the results, the perfect 100% solvation can be observed in the networks with charge $\delta_{0}$ = −0.6 to −1.0 and partial solvation for charge −0.4. The value of $\varepsilon_{r}$ is probably too low.

We can find the $\varepsilon_{r}$ = 57, where the analytical model would best fit the data from our molecular dynamics simulation assuming that the value $\varepsilon_{r}$ is constant. It can be interpreted, that the organic phase in dry network has some influence on the electrostatic potential, however, it is not so important as the influence of compact graphite plane. It remains still question for further investigation, what is exactly the value $\varepsilon_{r}$ in real materials. In reality, it can depend on the distance from macromolecule. In that case, it could have influence on acceleration of water molecules in the vicinity of macromolecular chains.

### 3.3. Water Molecules Bridging Two Macromolecular Chains

The presence of a solvent in the vicinity of the single chain is sufficient parameter, which is formally in qualitative correlation with a mechanical response. In this section, we present a detailed mechanism of how the solvation hangs together with viscoelasticity. We were interested in whether the water can change the topology of the network. It should form some temporary crosslinks between the macromolecular chains (water bridges). The presence of water molecular bridges has for a long time been presumed, for example, in humic acid in soil organic matter [29,30]. The topology of the water molecular bridges is described as a cluster of water molecules with specific molecular topology. However, the mechanism of action of a water bridge is probably more complex.

The mechanism is that solvation adds some temporary crosslinks to the network. It seems that there is the qualitative difference in the absence or presence of water bridges: network with partial charge −0.6 (0 bridges) and −0.8 (8 bridges). However, the quantitative relations between water bridges do not fit the macroscopic deformation response. The large change of behavior in Figure 2 between $\delta_{O}$ = −0.6 and −0.8 would be caused by 8 water bridges. The small change of macroscopic properties between $\delta_{O}$ = −0.8 and −1.0 should be caused by an increase of water bridging molecules from 8 to 46.

The second way of the calculation of the water bridges is based on a different principle. We do not classify the water molecules according to the distance of water and macromolecule. For each molecule of water in the simulation box, we found the first and the second nearest macromolecule and particularly nearest monomeric unit on both chains. Selected water molecules are considered bridges between the macromolecules when they satisfy next conditions.

1. The water must oscillate between two chains. The first and second nearest chains of particular water molecule must be the same during the entire simulation. They can alternate the position of nearest and the second nearest chain during the simulation, which is often the case.
2. The water molecule must be in the vicinity of a certain monomeric unit or near the neighboring monomer during the simulation. Even when the water molecule switches to another chain and returns, it must return to the vicinity of the same or neighboring monomeric unit.

The bridges can have a long distance. It was found that such water molecules were detected even at a distance of 0.75 nm from the macromolecule. From a distance of 0.8 nm, the molecules satisfying the criteria were not detected.

We found a number of such dynamic water bridges in the system. In our last article [24], we have proposed the structural parameter: ratio covalent/number physical crosslinks: cpc. It is a number of covalent crosslinks related to the number of physical crosslinks. The ratio is an intensive property, which does not depend on the size of the system. The numerical value gives information which interaction prevails. Analogously, we recalculated the number of water bridging molecules related to the number of physical crosslinks (wpc). Now, the acting can be compared to the covalent crosslinks from that article. The difference is that the covalent crosslinks are permanent, but the water crosslinks are separated. The values of wpc are presented in Table 2.

The deformation was described from the point of view of the model structure. The same reality can be analogously described from the point of view of mechanics. The temporary crosslinks in mechanics are characterized by their relaxation time, which is the average time for crosslink dissociation. This characteristic is important for the prediction of viscoelasticity and viscosity. The influence of relaxation is observed when the relaxation of crosslinks exceeds the time of deformation. The temporary crosslink must act as a bridge during the entire deformation. In that case, the relaxation of water bridges can be calculated by a correlation function.

**Figure 7:**
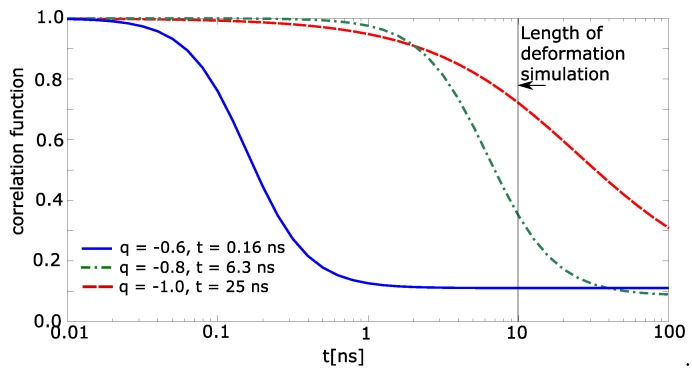
Relaxation of water, correlation function of water bridge (fraction of water persisting in water bridge) as a function of time; *q*—partial charge, *t*—average relaxation time. The relaxation times for −0.4≤q≤0 approach zero.

The correlation function was calculated for each individual molecule of water. It sets to the water molecule value 1 when the molecule meets the above-stated conditions (items 1 and 2). Otherwise, the correlation function is 0. The correlation function of simulation box was calculated as average value from all molecules of water, which were in the beginning near two macromolecular chains (nearer than 0.7 nm). The correlation functions are shown in Figure 7. The simulated time of deformation is 10 ns. The relaxation time for charge −1.0 exceeds the simulation time. The correlation function from Figure 7 was extrapolated by Boltzmann sigmoid. The limits of Boltzmann sigmoid were set fixed: initial top value of correlation function = 1.0; final bottom value = 0.1. The extrapolated parameters were mean logarithm of time, and slope of Boltzmann sigmoid.

It was found that the relaxation time for the charge −0.6 is 0.16 ns (Figure 7), for charge −0.8 time it is 6.3 ns, and for charge −1.0 the time is 25 ns. Thus, one can conclude that until the charge −0.6, the relaxation time is lower than the time of deformation and above −0.8, the relaxation time is comparable or exceeds the time of deformation. That presumes that the networks with partial charge −0.8 and −1.0 will be viscoelastic, which was really observed in Figure 2.

## 4. Discussion

There are cases where the solvation influences the deformation properties of materials [19,31]. The solvation is important for hydrogel networks and water [32]. The changes in deformation response induced by solvation have steep change at certain activity of water. The hydration offers an effective and comfortable way of adjusting of hydrogel properties. The existing structure of physical network can be conserved and one can modify only the response of hydrophilic part of network. For the description of the process, we need to know: i. control of solvation; ii. correlation between solvation and deformation response; iii. mechanism of deformation.

The first task is an adjustment of solvation without changing the character of physical network. We assume that the charge on flexible chain can be a structural parameter related simply to solvation. One can influence the charge by modification of the composition of macromolecular chains by different interacting groups in the chains or side groups [33]. The alternative is a modification of charge in solvent [34]. The advantage of this approach is that we can calculate the partial charges of atoms on chains. We do not need to know much data about the material. We need to know the composition and then we are able to calculate partial charges. The most available tool for the calculation of partial charge is the Extended Hückel analysis. The partial charges by extended Hückel analysis were calculated by software ChemBioOffice 3D version 13.2.3021 (Cambridge Soft, Cambridge, Great Britain). The hydrophilic chains in our model were composed of poly(ethylene glycol) (PEG). We calculated the partial charges in real poly(ethylene glycol). The positive charge on $CH_{2}$ group was (+0.175 e) and (−0.35 e) on oxygen. The value e is an elementary charge. The extended Hückel analysis probably underestimates the absolute value of charge on the chains. The real charges on PEG are probably ($\delta_{CH_{2}}$ > 0.175 e) and ($\delta_{O}$ < −0.35 e). The partial charge can also be calculated by Mulliken [35] population analysis. The calculation is more exact; however, it needs specific software for quantum chemical calculations.

The data can be probably generalized to any combination of solvent and network. The result of the analysis is information, whether the network will be solvated. It will give qualitative data, whether the solvent can contribute to the viscoelastic character of hydrogels or other materials with adsorbed water. For the polymers, the range of accessible partial charges is relatively broad from −0.35 poly(ethylene glycol) to −0.75 in proteins. Significantly larger charges (<−1) can be reached by dissociation of the ionic bond but, in this case, the electrostatic equilibrium also influences the acid-basic equilibrium. The partial charges are adjustable input values of the molecular dynamics simulations. In our model, we setup usually a partial charge for poly(ethylene glycol) calculated from the extended Hückel analysis. In contrast to real PEG, the partial charge on the model flexible hydrophilic chains can be adjusted can be scaled to arbitrary value. Model molecular chains presented in this article have a variable charge on oxygen from 0 to −1. Such a range is larger than the usual charges in real materials.

**Figure 8:**
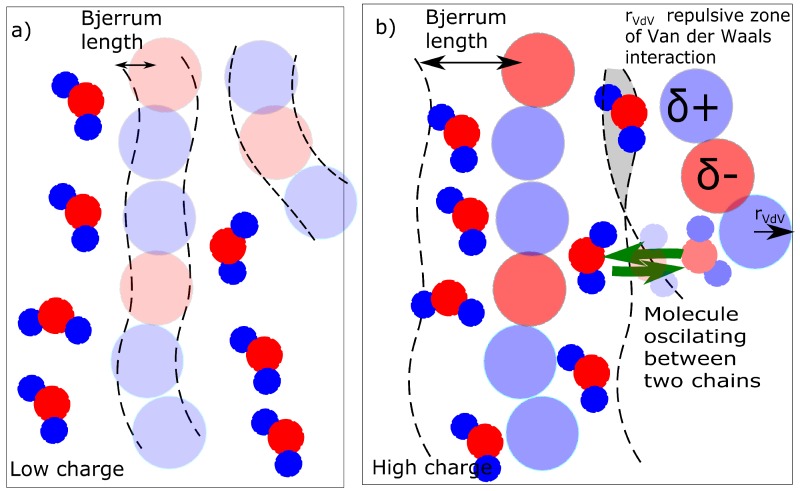
Solvation of macromolecular chain (**a**) low charge on chain-nonsolvated chain; (**b**) high charge (solvated) chain. Volume Inside circles-zone of van der Waals repulsion, dashed: Zone of solvation by Bjerrum length.

The result is a percentage of water molecules in the solvation zone delimited by Bjerrum length (Equation (3)). We describe the solvation simply as a concentration of molecules that are in the solvation zone of chains as described in Figure 8. If the Bjerrum length is within the repulsive radius of Van der Waals interaction, the water molecules are repulsed. The solvation zone is almost without solvent (Figure 8a). In the case of a high charge, the Bjerrum length is within the attractive zone and the water molecules are stabilized near the chains (Figure 8b). The mechanism of action of the charge was investigated from two aspects. The first aspect is a mixing energy, which can be calculated from the simulations. The first result is a detection of the water molecules in solvation zone of the molecular model (Figure 5b) as a function of partial charge.

The second alternative is analytical calculation of the solvation. In the analytical model, it can be calculated directly from the partial charge of the chain (Equations (5)–(8)). The results from analytical calculation (Figure 6) have the same trend as in Figure 5b (from the numerical molecular model) but are not numerically equal. Until the partial charge −0.5, we did not detect any solvated molecule neither in analytical nor in molecular model. The molecular model network with partial charge −0.6 contained 1% of water according to molecular dynamics simulation (0% of water molecules by analytical model). We detected 6% of solvated water molecules for a charge of −0.8 (15% by analytical model) and 17% of solvated water molecules in the simulation box for a network with a charge of −1.0 (100% by analytical model). The difference in numerical results between simulation and analytical model are expectable. In the molecular model, there is an excess of water molecules in the simulation box. The solvation zone of the molecular model can be saturated with a certain number of molecules and the other molecules are in bulk. It is important that in both cases we detect a change in network character between charges $\delta_{O}$ = −0.6 and −0.8.

The next alternative criterion is the presence of bridging molecules. Whether the water molecules bridge two chains and create a temporary ionic crosslink was investigated. The bridging can be investigated in two modes. One mode is the actual presence of a bridging molecule when one molecule of water is in the solvation zone of two chains (Figure 8b - gray field). The second mode is dynamic when one molecule of water is shared by two macromolecules and it oscillates between two macromolecules (Figure 8b - green arrows). The detailed models of solvation (water bridges) are discussed later in this article.

The second aspect is a relation between solvation and deformation properties. The experimental studies of charge and deformation response are not frequently presented. The reason is an interference of solvation and other effects such as pH-responsive behavior [6,7]. The model presented in this article enables us to keep the influence of pH constant and change only solvation degree on hydrophilic chains. The limit deformation properties, in which we observe the sharp change, are between the partial charges on oxygen on poly(ethylene glycol) chain $\delta_{O}$ = −0.6 and $\delta_{O}$ = −0.7. The $\delta_{O}$ = −0.6 is the highest charge of the network still with small hysteresis Figure 2 and $\delta_{O}$ = −0.7 is the lowest charge with hysteresis (Similar curve for the charge 0.667, Figure 4b). A solvation was a structural property analogously changed in the range of partial charges ($\delta_{O}$ is from −0.6 to −0.7). For the knowledge of factor which makes the difference, we must find structural factors between charge −0.6 and −0.7. The self-diffusion of water, which changes gradually in entire range of the charges (Figure 5a), is not the right factor. It was found that the hysteresis is observed when there are present atomic groups with a high charge and solvation of chains.

A correlation with mixing energy was investigated. The absolute value of mixing enthalpy is not in correlation with deformation response. The positive mixing energy means destabilization of system by solvent. The negative enthalpy means the stabilization. The switch from the positive and negative mixing enthalpy is observed in the range from −0.4 to −0.6. Another case is a course of mixing energy during deformation Figure 3, which has two alternatives a and b. The case a means thermodynamic stabilization of network, where the deformation energy of hydrogel is compared to partial energies of dry network and water from the same simulation. The case b means the influence of solvent to the structural evolution of network. The deformation energy of hydrogel is compared to the other simulations: deformation of dry network and deformation of box of water. The low-charge networks in-between $\delta_{O}$ = 0.0 and $\delta_{O}$ = −0.6 have similar a course of $\Delta W_{mix}$ in the case a and b in Figure 3. Both the influences in a and b are almost identical. That means the solvent influences the structural response only by thermodynamic stabilization or destabilization of the network. In the case of high charge $\delta_{O}$ = −0.8–1.0, the mixing energy functions are different. Probably, the most significant effect is the stabilization of polymer chains in micelles. Then the unfolding can be observed. The macromolecular network contributes to deformation by dissociation of interacting groups from micelles. Then the re-attachment is relatively difficult and slow. These effects are observed in the swollen state and not in dry networks.

The question is why the solvation is not in accord with absolute value of mixing energy. In fact, there are some networks (for example $\delta_{O}$ = −0.6) which are thermodynamically stabilized by mixing enthalpy and it is not reflected in solvation. The network with partial charge −0.6 has a mixing enthalpy of −8.3 MJ·mol^−1^. The number is relatively large because it is energy related to the mole of simulation boxes. The water is 65 weight % of the box and one box contains 2447 molecules of water. The relative mixing energy related to one mole of water is −2.2 kJ·mol^−1^. The thermal energy for the temperature 300 K is (*E* = RT) 2.5 kJ·mol^−1^. These calculations give us an insight into the relation of solvation—deformation—partial charge. The pure mixing enthalpy stabilizes the hydrogel solvent; however, the negative mixing enthalpy is not sufficient. The attractive energy must be even higher than the thermal energy of water. In this way, we can find out whether the water solvation will influence the deformation response of real materials. One can measure the mixing enthalpy by the calorimetry of any system macromolecule-solvent [36] or predict it from theory [37]. As well, the thermal energy of solvent can be calculated from temperature and Boltzmann constant. Then the structural changes in solvation and deformation can be predicted.

The third aspect is a mechanism of acting of solvent to the deformation properties. It is presumed that the water must modify the structure. In this article, we examine the hypothesis that also in hydrogel the water molecule can be shared by two macromolecules and acts as the bridge. The presence of water molecular bridges is discussed in humic acids of soils [30]. The reason why they are not detected in hydrogels is the excess of water. It is very difficult to detect the water molecules with different relaxation behavior than the normal water molecules. Nevertheless, there was experimentally detected some strongly bonded water also in hydrogels [2]. The strong interaction is reflected by the relaxation of the water. Similarly, the strongly bonded water has influence on relaxation in models (Figure 7).

The question, whether the water molecular bridges must reflect interaction of organic part and water. It is relatively weak to cause significant changes in deformation response. The structural change reflecting deformation behavior was concerning the water in the vicinity of the macromolecule. The first model is that the water is considered a bridge when it is simultaneously in the solvation zones of two macromolecular chains. In that case, the structural difference between the samples is significant. The bridging water molecules are not present up to a partial charge of $\delta_{O}$ = −0.6. At the partial charge of $\delta_{O}$ = −0.8, there were found 0.3% of bridging molecules (8 water molecules from 2447) and for the network with a partial charge $\delta_{O}$ = −1.0, 1.9% of all molecules (46/2447) are bridging. The water molecules bridging two macromolecular chains can in principle contribute to the change of behavior because they increase the network density. The qualitative change is between $\delta_{O}$ = −0.6 and −0.8; however, it is not in correlation quantitatively. The large change of behavior in Figure 2 between $\delta_{O}$ = −0.6 and −0.8 would be caused by 8 water bridges. The next small change of macroscopic properties between $\delta_{O}$ = −0.8 and −1.0 should be caused by an increase of water bridging molecules from 8 to 46. In that mechanism, the change in hysteresis would be expected rather between $\delta_{O}$ = −0.8 and −1.0.

**Figure 9:**
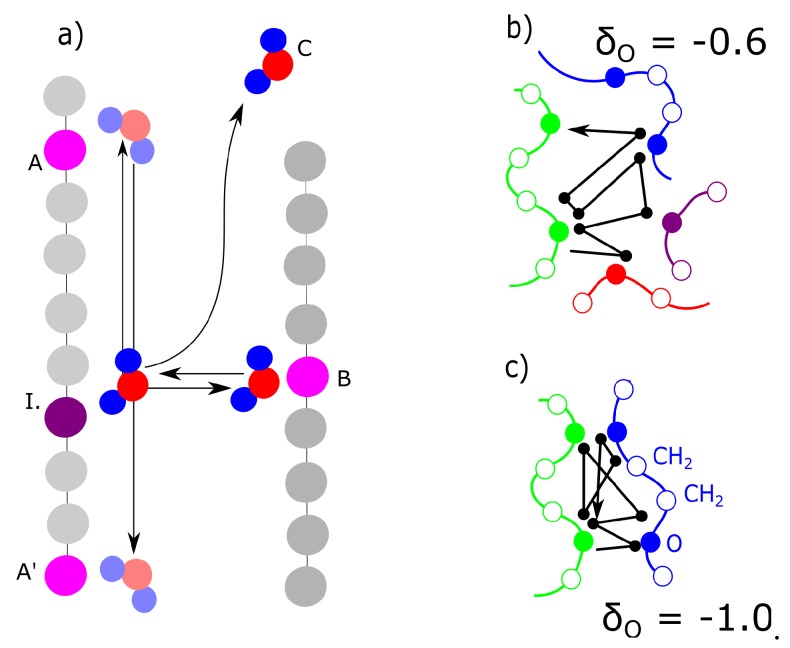
Mechanisms of delay of water in the vicinity of macromolecules. (**a**) schematic picture I—delay in the vicinity of single atomic group; A,A’—delay near single chain, but moving along the chain; B—oscillation between two chains; C—dissociation from chain; (**b**) trace of water in the network with moderate partial charge; (**c**) trace of water at high charge, delay of water between two chains and neighboring monomeric units.

The actual concentration of bridges does not have probably sufficient correlation with deformation response. The bridges are probably dynamic. The criteria for dynamic water bridges are described in Figure 9a. We detected the nearest and second nearest macromolecule for each molecule of water. Some water molecules are present in vicinity of one macromolecule: position (I)—initial position. They switch to the opposite position on the second chain is marked as B. The cases in Figure 9a were compared from the criteria as to how frequently they were observed. The case, when a molecule delayed in an initial position (I), was never observed in any model. The case (C) was observed in general relatively frequently; however, it was excluded from analysis. We selected for analysis only the water molecules which were attached to at least one macromolecule during the entire simulation. Case (A) or (A’) is movement along the chains. Both the networks with a moderate ($\delta_{O}$ = −0.6) and high ($\delta_{O}$ = −1.0) partial charge showed movement along the chains with similar intensity. In both networks movement to a neighbor or second neighbor was observed. In this aspect, we did not observe any difference between the networks with moderate partial charges and those with high partial charges.

As stated in the previous paragraph, uniquely the molecules of water, switching between initial position I. and opposite position B. can be considered bridges. Their number changed between charge −0.6 and −0.8. For the charge −0.6, the water also shifts mostly to the nearest-neighbor monomer, but it oscillates mostly between 3 chains. Some individual water molecules oscillated in the vicinity of 2 or 4 macromolecules (Figure 9b). In the case of the network with a high charge of −1.0, the molecules oscillated between two chains (Figure 9c). The connection in Figure 9c can, in fact, be considered a crosslink, which connects two chains. It can be an interesting structural component, as it is not as rigid as, for example, a covalent crosslink. On the other hand, at a low charge (−0.6), it is relatively weak and cannot be considered a crosslink. It is interesting that some molecules, which form the bridge are relatively distant from macromolecule. The electrostatic interaction is acting for long distances.

The water bridges from Figure 9c were quantified. The basic output of the model was a concentration of water bridges per simulation box. We would like to compare the acting of the water bridges and acting of another type of interactions. We presented elsewhere an analogous study [24]. The covalent crosslinks were added to the physical network instead of water bridges. The effect of covalent crosslink is significantly different from the effect of water bridge. However, one can compare the intensity of interactions. When certain concentrations of covalent crosslinks do not have any effect on the physical network, the same number of water bridges will not have the effect as well. We have proposed the structural parameter: ratio covalent/number physical crosslinks: cpc. Analogously, we propose wpc in this article: ratio water bridges/number physical crosslinks: wpc.

The wpc is a function of partial charge on the flexible chains: wpc(δ). The networks with charges of −0.4 to 0.0 have no bridging molecule $wpc_{\left(\delta_{O}\leq -0.4\right)}$ = 0. It is equivalent to physical network cpc = 0. All the networks show moderate hysteresis. There was expected no change between behavior of physical network and such networks.

The networks with partial charge −0.6 charge has $wpc_{\left(\delta_{O}=-0.6\right)}$ = 0.39 ± 0.04, where the water bridges are in the minority. The average value and standard deviation were calculated from 4 independent models. That has almost zero influence on the deformation properties. It can be compared to the networks with cpc = 0.35, where the influence of deformation behavior was zero, and cpc = 0.7, where the deformation response changed only slightly.

Network with partial charge −0.8 has $wpc_{\left(\delta_{O}=-0.8\right)}$ = 2.1 ± 0.3. The behavior changes significantly to viscoelastic properties. A significant change of was observed also in the case of covalent crosslinks from cpc = 1.45.

The network with a partial charge of −1.0 has $wpc_{\left(\delta_{O}=-1.0\right)}$ = 4.1 ± 0.2. The number of water connections is significantly higher than the number of physical crosslinks. The effect of water bridges is similar to a moderately crosslinked network from the previous paragraph. On the other hand, the strong covalent crosslinking changes the response qualitatively. The cpc = 3.05 to 6.5 lead to an elastic response.

It seems that the covalent crosslinks and water molecular bridges have opposite effects. The original physical network without any additive interaction has a moderate hysteresis of deformation response. The addition of covalent crosslinks changes the response to elastic deformation. The water molecule bridges change the behavior rather to a viscoelastic response. The similarity of covalent crosslinks and water bridges is that they have small influence at low concentration. For the change of behavior due to covalent crosslinks we need three times more covalent molecules than physical crosslinks in order to reach the change. In the case of water bridges, we need to have at least 2 water bridges per one physical crosslink. The water bridges are specific in their delocalization. When they are dissociated, they can be re-created at another place. It is reflected in increased viscoelasticity.

## 5. Conclusions

The viscoelastic properties of hydrogels depend on the solvation of macromolecular chains of the hydrogel network. The solvation can be investigated by a molecular model, where all other factors except the degree of solvation are set at a constant. The solvation of hydrophilic chains can be regulated by a partial charge on the chains. The solvation was evaluated by the Bjerrum length. The network chains in the model are surrounded by a zone of solvation, whose thickness is equivalent to the Bjerrum length (BL). This is the zone where the attraction of the solvent is higher or equal to the thermal energy (kT). When the partial charge on the atom is too low, the BL is low and the molecules of solvent are repulsed from the solvation zone. The network, in that case, shows low hysteresis. On the other hand, when the partial charge on the chain is high, some of the molecules of water are attracted to the solvation zone and the deformation behavior of the network shows hysteresis. The hysteresis always hangs together with charges, which must be sufficiently high. It does not depend on the total average density of the charge in the box. Even a box with a small charge density shows hysteresis when it contains a certain number of highly charged atomic groups. The next aspect is the presence of water molecules bridging two macromolecular chains. In this case, the presence of such molecules in solvation zones is not a sufficient criterion. The number of such molecules permanently persisting in solvation zone and shared by two chains is very low. A more appropriate criterion is the oscillation of water molecules between two chains and particularly to atomic groups and their neighbors. The persistence of water molecules near two macromolecules is detected by relaxation time. The model can be applied to the design of properties of hydrogels. The model enables one to separate the influence of solvation from the effect of acid-basic interaction. The solvation can be analyzed without making significant changes in micellar or network structure. In real macromolecules, one can increase and decrease charge by the composition of the macromolecular network. It enables one to regulate the deformation response of the real material. 

## Figures and Tables

**Table 1:** List of parameters of simulations.

Parameter	Value
**Single input variable in this article, other parameters are constant**	
Partial charge on oxygen in PEG chain (e—elementary charge unit)	<0 e, −1 e>
**Properties of simulation**	
Number of PEG chains	8
Number of monomeric units in each chain: 1 monomer = 3 beads (CH_2_–CH_2_–O)	60
Number of interacting AA groups forming the physical crosslinks in each chain	20
pH	Acidic
Interaction energy between AA groups at given pH	25 kJ mol^−1^
Interaction energy PEG-PEG	0.34 kJ mol^−1^
Interaction energy PEG-WATER	0.67 kJ mol^−1^
Temperature	300 K
Box size (in undeformed state)	5 nm
**Particles present in the simulation box**	
PEG—$CH_{2}$ atomic groups (partial charge dependent on PEG oxygen)	960
PEG—*O* atomic groups (partial charge variable)	480
PEG—interacting AA—Acrylic acid group (Partial charge 0)	160
WATER Hydrogen (partial charge 0.425 e)	4894
WATER Oxygen (partial charge −0.85 e)	2447
**Molecular dynamics method**	
Software GROMACS/ensemble	NVT
Standard leap-frog md integrator	md
Thermostat	V-rescale
Electrostatic interaction—Particle-Mesh Ewald electrostatics.	PME
Type of deformation	Shear
Amplitude of deformation	100%
Deformation rate	0.1 ns^−1^

**Table 2:** Presence of water bridges in networks; wpc- water bridges related to number of physical crosslinks.

Charge	wpc
−0.4 to 0.0	0.0
−0.6	0.39±0.04
−0.8	2.1±0.3
−1.0	4.1±0.2

## Abbreviations

The following abbreviations are used in this manuscript:

BL	Bjerrum length—physical property of material
cpc	ratio of covalent and physical crosslinks in the model network—structural property of hydrogel network
wpc	ratio of water bridges and physical crosslinks in the model network—structural property of hydrogel network
MD	molecular dynamics—method
PEG	poly(ethylene glycol)-component of polymer network, IUPAC name: poly(oxyethylene)
OW	Oxygen atom—specific symbol for oxygen in water (when it must be distinguished from oxygen in PEG: O)
